# Renal biopsy in patients over 75: 131 cases

**DOI:** 10.5414/CN108258

**Published:** 2014-08-27

**Authors:** Cristiana Rollino, Michela Ferro, Giulietta Beltrame, Giacomo Quattrocchio, Carlo Massara, Francesco Quarello, Dario Roccatello

**Affiliations:** Nephrology, S.G. Bosco Hospital, Turin, Italy

**Keywords:** elderly, renal biopsy, glomerulonephritis, renal disease

## Abstract

Introduction: Demographic analysis shows the ageing of the global population and the consequent increase in the age of hospitalized subjects and of patients starting dialysis. Hence, interest in the feasibility, safety, and usefulness of renal biopsy in elderly patients is growing. We examined the data of 131 patients over the age of 75 who underwent renal biopsy. We analyzed the safety of the procedure, treatment, and outcomes. Results: Histological diagnoses included: membranous glomerulonephritis (GN) 20.6%, crescentic GN 12.9%, IgAGN 10.6%, focal segmental glomerulosclerosis 9.1%, acute GN 4.5%, amyloidosis 9.1%, and acute tubular necrosis 3.8%. Mean glomerular obsolescence was 28.9 ± 27.9%. Mean age of the patients was 78.7 ± 5.73 years. At the time of biopsy, serum creatinine (SCr) was 4.47 ± 2.56 mg/dL and proteinuria was 4.82 ± 6.78 g/day. Targeted treatment was given to 51.9% of patients, 52.9% of whom had a good clinical response. Eight patients had clinically non-relevant side effects (11.7%). A positive response (defined as a more than 50% reduction of SCr, or by partial or complete remission of proteinuria) was observed in 36 patients (52.9%). 76 patients were monitored for 57 ± 9.89 months: 18 patients were on dialysis (follow-up 2.56 ± 3.61 months), 15 died (follow-up 58.5 ± 13.43 months), and 52 remained under nephrologic observation for 36 ± 31 months (SCr was 2.56 ± 0.75 mg/dL and proteinuria was 4.82 ± 6.78 g/day). Conclusion: In our experience, renal biopsy is safe even in very elderly patients; it allowed targeted treatment in 51.9% of patients, 52.9% of whom had a good clinical response, possibly contributing to prolonged patient survival and improved quality of life.

## Introduction 

Demographic analysis shows the ageing of the global population (in Italy people over 75 represented 10% of the population in 2010 and 8.3% in 2002) [1, 2] and the consequent increase in the age of hospitalized subjects [3] and of patients starting dialysis [4]. 

Despite the increasing age of the dialysis population, little information is available about the causes of renal diseases in older patients. 

Renal biopsy plays a central role in diagnosing causes of acute renal failure with uncertain etiology and glomerular diseases, which in the elderly are represented most commonly by minimal change disease, membranous glomerulopathy, crescentic glomerulonephritis (GN), and amyloidosis [3, 5, 6]. 

To ascertain the feasibility, safety, and usefulness of renal biopsy in elderly patients, we retrospectively examined the data of 131 patients over 75 years of age who underwent renal biopsy. They represent 11.1% of the 1,178 renal biopsies performed at our center between 1974 and January 2012. 

## Methods 

Our Nephrology Unit serves a population of ~ 500,000 inhabitants in Turin located in north-western Italy. The hospitalization rate in the nephrology ward is ~ 500 patients a year. 

At our center, we performed 1,178 renal biopsies between 1974 and January 2012. 

We selected 131 patients (11.1%) over 75 years of age from the entire cohort and examined the complications of the biopsy procedure, histological diagnosis, treatment, and the outcome of the patients. 

### Renal biopsy

Renal biopsy is performed with real-time ultrasound (US) guide, using automatic 18 gauge, 15 or 22 cm Tru-Cut needles. Not more than two punctures are made. One patient underwent surgical biopsy. 

Feasibility criteria for undergoing renal biopsy include: kidney size > 8 cm at US examination, bleeding time according to Ivy test < 10 min, platelet count > 100,000/mm^3^, Hb > 10 g/dL, and normal coagulation parameters (prothrombin and thromboplastin time). 

Patients remain in bed for 24 hours following the procedure. Vital parameters are monitored every hour for the first 4 hours and then every 2 hours. US and renal echo-Doppler are performed the day after in order to exclude hematomas and arteriovenous fistulas. Patients remain in hospital for 2 days after the procedure. 

## Results 

### Biopsy procedure

The biopsy sample allowed diagnosis to be made in all cases. The mean number of glomeruli was 15.9 ± 8.5 per biopsy sample. In 89.2% of samples, there were more than 8 glomeruli. Mean glomerular obsolescence was 28.97 ± 27.9 (0 – 82%) of the total number of glomeruli present in the biopsy sample. 

### Patients

Mean age of the 131 patients (72 men, 59 females) was 78.7 ± 5.73 (75 – 93) years. 

At the time of biopsy, serum creatinine (SCr) was 4.47 ± 2.56 mg/dL and proteinuria was 4.82 ± 6.78 g/day. 

### Biopsy safety

Minor complications were observed in 11.4% of cases. They consisted of hematoma < 2 cm in 6 patients, hematoma > 3 cm in 2 patients, arteriovenous fistulas in 3 patients, hematuria in 3 patients, and pseudo-aneurysm communicating with a renal cyst in 1 patient. 

The 2 patients who developed large hematomas included a 75-year-old woman on dialysis because of crescentic GN who presented severe bleeding into the perirenal space, and a 76-year-old man with membrano-proliferative GN, with small kidneys (8.5 cm) undergoing renal biopsy for the second time, who presented severe bleeding into the retroperitoneum requiring arterial embolization. 

### Histological diagnoses ( Figure 1 )

**Figure 1.**
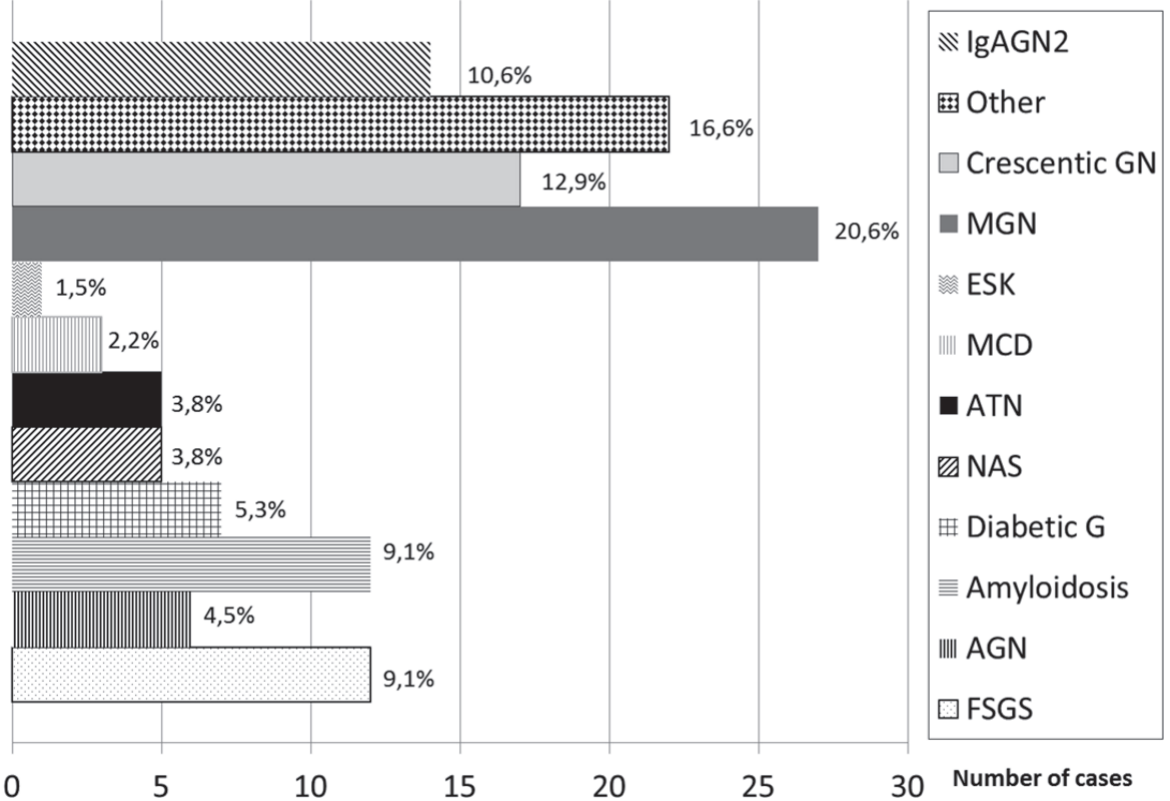
Main histological diagnosis. IgAGN = IgA glomerulonephritis; MGN = membranous glomerulonephritis; ESK = end-stage kidney; MCD = minimal change disease; ATN = acute tubular necrosis; NAS = nephroangiosclerosis; Diabetic G = diabetic glomerulosclerosis; AGN = acute post-infectious glomerulonephritis; FSGS = focal segmental glomerulosclerosis. The complete list of diagnoses is: MGN in 27 patients (20.6%), crescentic GN in 17 (12.9%; ANCA-associated in 11 cases, cANCA in 2 and pANCA in 9, including anti-glomerular basement membrane in 2), IgAGN in 14 (10.6%; in 1 with cholesterol embolism), FSGS in 12 (9.1%; in 1 with cholesterol embolism), AGN in 6 (4.5%), amyloidosis in 12 (9.1%; 7 AL, 5 AA), diabetic G. in 7 (5.3%), NAS in 5 (3.8%), ATN in 5 (3.8%), thrombotic microangiopathy (TMA) in 3 (2.2%), MCD in 3 (2.2%), membrano-proliferative GN (MPGN) in 4 (3%) (in 2 cases secondary to cryoglobulinemia), light chain disease (LCD) in 3 (2.2%), non-specific changes in 2 (1.5%), cholesterol embolism (CE) in 1 (0.7%), cast nephropathy in 2 (1.5%), ESK in 2 (1.5%), acute interstitial nephropathy in 1 (0.7%), sarcoidosis in 1 (0.7%), IgMGN in 2 (1.5%), mesangial GN in 1 (0.7%), and immunotactoid GN in 1 (0.7%).

Histological diagnoses of the whole series are reported and specified in Figure 1. The most frequent diagnoses were membranous glomerulonephritis (MGN) in 27 patients (20.6%), crescentic GN in 17 (12.9%; ANCA-associated in 11, cANCA in 2, and pANCA in 9 cases, including anti-glomerular basement membrane in 2), IgAGN in 14 (10.6%; 1 with cholesterol embolism), focal segmental glomerulosclerosis (FSGS) in 12 (9.1%; 1 with cholesterol embolism), acute post-infectious GN in 6 (4.5%), and amyloidosis in 12 (9.1%; 7 AL, 5 AA). 

### Treatment ( Figure 2 )

68 patients (51.9%) were treated with corticosteroids and/or immunosuppressive drugs (65 patients), immunoglobulins (2 patients) or colchicine (1 patient) on the basis of histological diagnosis and clinical situation: 3 minimal change disease, 11 crescentic GN, 18 MGN, 6 FSGS, 5 IgAGN, 3 acute post-infectious GN, 3 light chain disease, 3 AA amyloidosis, 5 AL amyloidosis, 3 membranoproliferative GN (in 2 cases secondary to cryoglobulinemia), 1 cast nephropathy, 1 cholesterol embolism, 3 thrombotic microangiopathy, 1 sarcoidosis, and 1 extrarenal vasculitis. 

Eight patients had side effects (11.7%) to treatment (Herpes zoster infection in 3 patients, pneumonia in 1, hallucinations in 1, leukopenia in 2, mucositis in 1). No severe side effects were reported in any of these cases. 

Response to treatment was defined as a more than 50% reduction of SCr, or by partial (0.5 – 3 g/day) or complete (< 0.5 g/day) remission of proteinuria. 

On the whole, response was observed in 36 patients (52.9%) who were followed-up for 59 ± 12 months (Figure 3 and Figure 4). Three patients had a relapse which was treated again (2 MGN patients were treated with cyclosporin A with response in 1, and 1 patient with FSGS treated with cyclosporin A and cyclophosphamide who responded). 32 patients (47.1%) did not respond (Figure 3). 

In detail (Table 1), we treated 18 patients with MGN: 15 were given a 6-month corticosteroid/immunosuppressive drug regimen at reduced dosage according to Ponticelli et al. [7]; 1 subject was treated with cyclosporin A, and 2 with high-dose immunoglobulins (400 mg/kg/day for 3 consecutive days) because of diabetes. At the end of follow-up, 6 patients had achieved complete remission. One patient died within 5 months, 1 within 6 months and another within 47 months. 

The 14 patients with IgAGN had moderate sclerotic changes (mean glomerular obsolescence 30%); crescents were observed in 5 patients. In 1 subject, IgAGN was associated with cholesterol embolism, in another with acute tubular necrosis, and in another one with nephroangiosclerosis. Corticosteroid treatment was given to 5 patients. 

Acute post-infectious GN was observed in 6 cases (4.5%) (Table 2), with 1 case each related to pneumonia, to urinary tract infection, to otitis, to fever which appeared after an anti-influenza vaccination, and to sialadenitis, while in the last case we could not detect any significant clinical elements. 

Crescents were observed in 2 patients. SCr ranged from 1.5 to 3.8 mg/dL and proteinuria from 0.9 to 3.8 g/day. 

### Outcome ( Figure 4 )

76 patients were followed for 57 ± 9.89 months: 18 patients were on dialysis (follow-up 2.56 ± 3.61 months), 15 died (including 9 on dialysis; follow-up 58.5 ± 13.43 months), and 52 remained under nephrologic observation for 36 ± 31 months (SCr was 2.56 ± 0.75 mg/dL and proteinuria was 4.82 ± 6.78 g/day at the end of follow-up). 

55 patients (41.9%) were lost during the follow-up period: 20 were referred to other hospitals, 8 died, 2 refused treatment, and 25 were lost. 

## Discussion 

Along with the ageing of the global population, we are faced with the kidney diseases typical of the elderly, such as acute renal failure secondary to pre-renal conditions, vascular nephropathies, and, among the glomerular diseases, crescentic GN, membranous GN and paraproteinemia-associated nephropathies [3, 5, 6]. For many of these conditions, renal biopsy is needed to start targeted treatment and to assess the extent of the renal sclerotic changes, which, together with the clinical conditions and the possible comorbidities, might suggest whether a treatment is suitable or not. 

Currently, no literature reports exist on a large series of renal biopsies carried out on patients older than 75 years of age. 

Our experience with renal biopsy in elderly patients is positive in regards to feasibility, safety, usefulness and outcome of the patients. 

The proportion of suitable biopsy samples in our series was very good, as diagnosis was possible in all cases. We obtained > 8 glomeruli per sample in 89.2% of cases, with 8 glomeruli being considered a benchmark for correct diagnosis [8]. 

Among the 131 patients aged more than 75 years representing 11.1% of the biopsies performed in our center between 1974 and January 2012, we observed minor complications in 11.4% of cases, which is similar to the frequency reported in the literature [9, 10]. Two patients presented severe bleeding into the retroperitoneum. One of these patients had very small kidneys (8.5 cm) and the other had crescentic GN with severely compromised renal function which required replacement therapy. 

Histological diagnoses were distributed according to the already described spectrum in this age range: membranous GN was identified in 20.6% of patients, crescentic GN (including 2 anti-glomerular basement membrane) in 12.9%, focal and segmental glomerulosclerosis in 9.1%, and amyloidosis in 9.1% (Figure 1). 

Interesting results regard the nephropathies which are considered less common in the elderly. IgAGN represented 10.6% of cases. A similar frequency (11.3%) was found by Moorthy et al. [11] in a series of patients older than 65 years. In an analysis of 235 patients aged more than 80 years, Moutzouris et al. [12] found a frequency of IgAGN similar to that found in the control group of younger patients (aged 60 – 81 years): 7.1% vs. 5.4%. In our experience, IgAGN represents 25.6% of nephropathies in patients younger than 75 years. 

Our elderly patients with IgAGN had moderate sclerotic changes (mean glomerular obsolescence 30%); crescents were observed in 5 cases. In 1 case, IgAGN was associated with cholesterol embolism, in another with acute tubular necrosis, and in another one with severe vascular disease. 

Acute post-infectious GN was found in 6 cases (4.5%) (2 with crescents). The literature indicates a frequent appearance of acute post-infectious GN in diabetic patients, e.g., 11.1% in the series by Pham et al. [13] with a mean age of 60.1 years, but we could find no reports regarding this issue in the elderly. In Moutzouris’ series [12], 4 patients out of 235 had acute post-infectious GN (1.7%) . 

**Figure 2.**
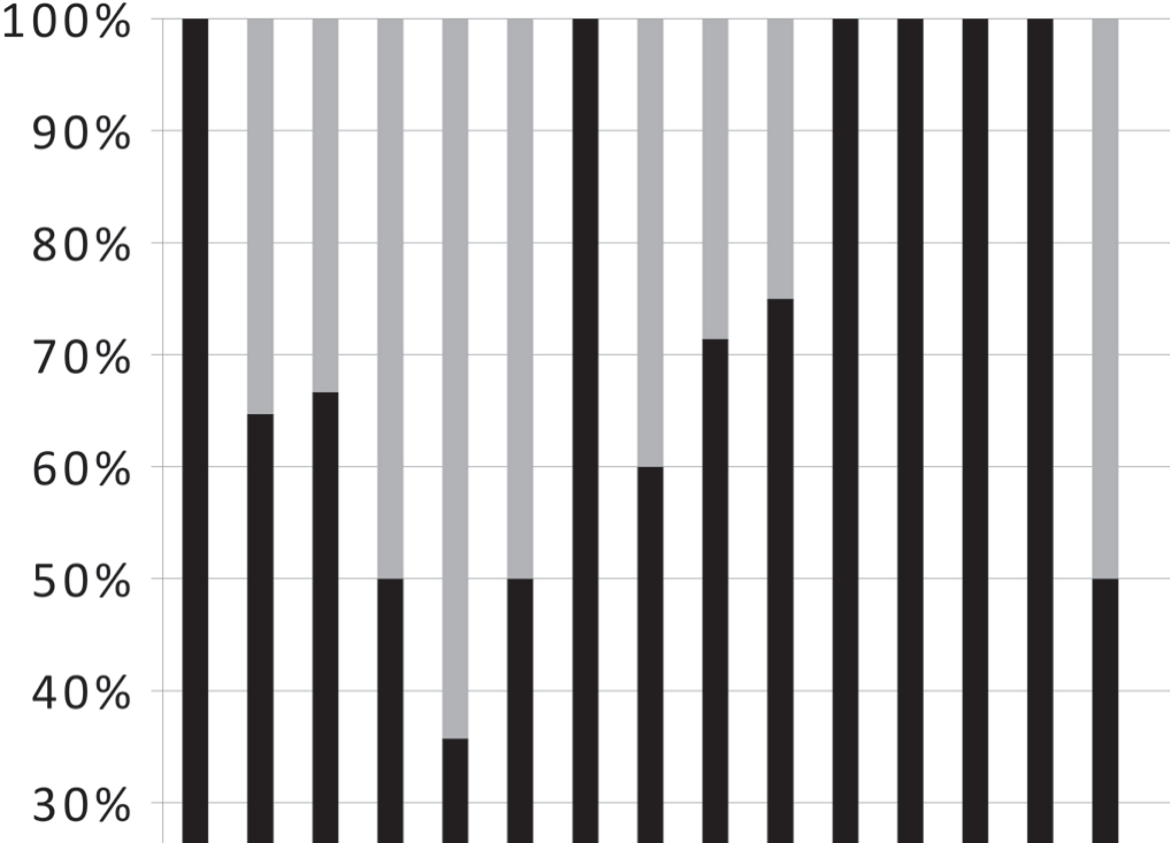
Treated patients. MCD = minimal change disease; crescentic GN = crescentic glomerulonephritis; MGN = membranous glomerulonephritis; FSGS = focal segmental glomerulosclerosis; IgAGN = IgA glomerulonephritis; AGN = acute post-infectious glomerulonephritis; LCD = light chain disease; AA amyl = AA amyloidosis; AL amyl = AL amyloidosis; MPGN = membrano-proliferative glomerulonephritis; CE = cholesterol embolism; TMA = thrombotic microangiopathy.

**Figure 3.**
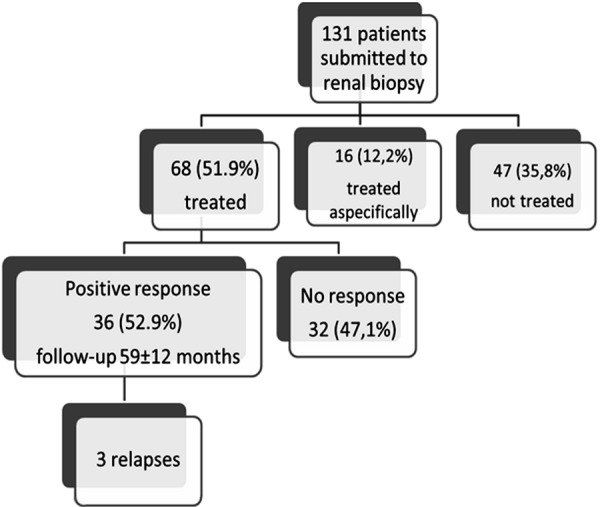
Treatment.

**Figure 4.**
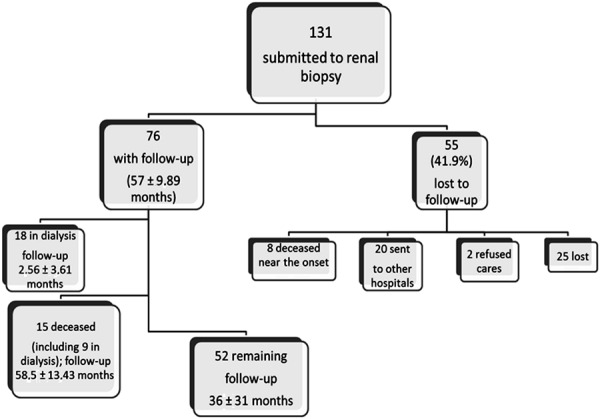
Follow-up.

Treatment with corticosteroids and/or immunosuppressive drugs, colchicine or immunoglobulins was given to 68 patients (51.9%) (Figure 2 and Figure 3). 36 of the treated patients (52.9%) had good clinical response consisting of either improvement in renal function or reduction or complete remission of proteinuria (Figure 3 and Figure 4). Moderate side effects were observed in 8 patients (11.7%). 

On the whole, we were able to follow-up 76 patients for 57 ± 9.89 months: 18 patients had end stage renal disease on replacement treatment, 15 died (including 9 in dialysis) after 58.5 ± 13.43 months, and 52 had a mean SCr of 2.5 mg/dL over a follow-up of 36 ± 31 months (Figure 4). 

In conclusion, the study covers a long period of time (38 years) and, considering how much epidemiology and treatments have evolved during this period, the outcomes may be different nowadays. This represents the limitation of our study. Nonetheless, it constitutes the largest single-center series of renal biopsies carried out on patients older than 75 years. The results show that renal biopsy is a safe procedure even in very elderly patients. It allowed us to administer targeted treatment in 51.9% of patients, 52.9% of whom showed good clinical response. Furthermore, it contributed to prolonging the survival of patients and improving their quality of life. 

## Conflict of interest 

No conflict of interest. 

**Table 1.** Treatment.

Diagnosis	Number	Treatment	Clinical response
MCD	3	CS (1), CS + CyA (1)	2
Crescentic GN	12	CS (2), CS + CyF (3), CS + CyF + PE (6)	9 (7 lost)
MGN	18	CS + Chi (15), lg (2), CS (1)	8 (6 lost)
FSGS	6	CS (4), CS + CyF (2)	3 (3 lost)
lgAGN	5	CS (4), CS + CyF (1)	3 (2 lost)
AGN	3	CS (3)	2 (1 lost)
LCD	3	CS + CyF (2), CS + VCR + A (1)	1
AA amyloidosis	3	Colchicin (1), CS + Chi, VAD	1 (1 died)
AL amyloidosis	5	MP(5)	1
MPGN	3	CS (1), CS + CyF (2)	1 (1 lost)
Cast nephropathy	1	MP + PE	lost
CE	1	CS	0
TMA	3	CS+ plasma (3)	3
Sarcoidosis	1	CS	0
Extrarenal vasculitis	1	CS	1

Clinical response = number of patients with clinical response to treatment; MCD = minimal change disease; Crescentic GN = crescentic glomerulonephritis; MGN = membranous glomerulonephritis; FSGS = focal segmental glomerulosclerosis; IgAGN = IgA glomerulonephritis; AGN = acute post-infectious glomerulonephritis; LCD = light chain disease; MPGN = membrano-proliferative glomerulonephritis; CE = cholesterol embolism; TMA = thrombotic microangiopathy; CS = corticosteroid; CyA = cyclosporin A; CyF = cyclophosphamide; PE = plasma exchange; Chl = chlorambucil; VCR = vincristin; A = adriblastin; VAD = vincristine + adriblastin + dexametasone.

**Table 2.** Patients with acute post-infectious glomerulonephritis.

Patient	Crescents/glom. scl.	lnfectious disease	SCr b mL/dL	SCr e mL/dL	Prot b g/day	Prot e g/day	Treatment	Follow-up
1	+/9%	Sialadenitis	1.5	1.5	2.6	1.4	Antibiotic	12
2	–/37%	Pneumonia	3.1	3.2	3.8	0.1	CS	88
3	–/23%	Otitis	2	4	0.9	1.6	–	86
4	+/0	Urinary tract infection	2		2.7		CS	Lost
5	–/11%	Fever after influenza vaccination	2.4		1		CS	Lost
6	–/6%	–	3.8	1.5	2.7	0.5	Antibiotic	30

Glom. Scl. = percentage of sclerotic glomeruli; SCr b = serum creatinine at biopsy; SCr e = serum creatinine at the end of follow-up; Prot b = proteinuria at biopsy; Prot e = proteinuria at the end of follow-up; CS = corticosteroid.
